# Tailoring Carbon Tails of Ligands on Au_52_(SR)_32_ Nanoclusters Enhances the Near-Infrared Photoluminescence Quantum Yield from 3.8 to 18.3%

**DOI:** 10.1021/jacs.3c09846

**Published:** 2023-11-20

**Authors:** Yitong Wang, Zhongyu Liu, Abhrojyoti Mazumder, Christopher G. Gianopoulos, Kristin Kirschbaum, Linda A. Peteanu, Rongchao Jin

**Affiliations:** † Department of Chemistry, Carnegie Mellon University, Pittsburgh, Pennsylvania 15213, United States; ‡ Department of Chemistry and Biochemistry, University of Toledo, Toledo, Ohio 43606, United States

## Abstract

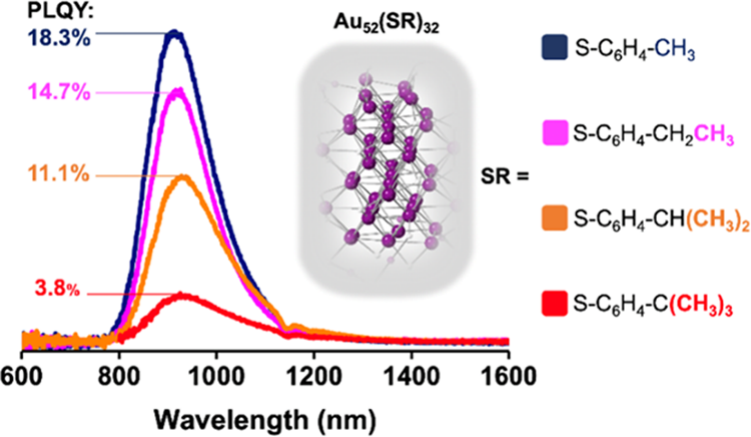

One of the important factors that determine the photoluminescence (PL) properties of gold nanoclusters pertain to the surface. In this study, four Au_52_(SR)_32_ nanoclusters that feature a series of aromatic thiolate ligands (–SR) with different bulkiness at the *para*-position are synthesized and investigated. The near-infrared (NIR) photoluminescence (peaks at 900–940 nm) quantum yield (QY) is largely enhanced with a decrease in the ligand’s *para*-bulkiness. Specifically, the Au_52_(SR)_32_ capped with the least bulky *p*-methylbenzenethiolate (*p*-MBT) exhibits the highest PLQY (18.3% at room temperature in non-degassed dichloromethane), while Au_52_ with the bulkiest *tert*-butylbenzenethiolate (TBBT) only gives 3.8%. The large enhancement of QY with fewer methyl groups on the ligands implies a nonradiative decay via the multiphonon process mediated by C–H bonds. Furthermore, single-crystal X-ray diffraction (SCXRD) comparison of Au_52_(*p*-MBT)_32_ and Au_52_(TBBT)_32_ reveals that fewer methyl groups at the *para*-position lead to a stronger interligand π···π stacking on the Au_52_ core, thus restricting ligand vibrations and rotations. The emission nature is identified to be phosphorescence and thermally activated delayed fluorescence (TADF) based on the PL lifetime, ^3^O_2_ quenching, and temperature-dependent PL and absorption studies. The ^1^O_2_ generation efficiencies for the four Au_52_(SR)_32_ NCs follow the same trend as the observed PL performance. Overall, the highly NIR-luminescent Au_52_(*p*-MBT)_32_ nanocluster and the revealed mechanisms are expected to find future applications.

## Introduction

Quantum-sized, atomically precise gold nanoclusters (NCs) exhibit intriguing photoluminescence (PL) owing to their nonmetallic nature and discrete electronic energy levels.^1^ The small gaps of such NCs (<1.5 eV) give rise to PL in the near-infrared (NIR) region, which holds potential in deep tissue bioimaging, sensing, photonics, and anticounterfeiting applications.^1−6^ However, highly luminescent Au NCs in the NIR region are still rare because the deactivation of the excited state tends to be dominated by nonradiative relaxation according to the energy gap law, resulting in low quantum yields in the NIR region (QYs of Au NCs often below a few %).^7^

To understand the origin of the deactivation of excited states in NIR-luminescent Au NCs, structural information is critically important. Crystallographic analysis revealed that thiolate-protected Au_*n*_(SR)_*m*_ NCs typically possess a core–shell structure, in which the inner Au(0) atoms form a stable core and the oligomeric –SR–[Au(I)–SR–]_*x*_ surface motifs (*x* = 1, 2, 3, etc.) form the shell.^7^ By adopting different synthetic methods, the core (or kernel) of Au NCs can be tailored and a rich library of structures such as the tetrahedron, cuboctahedron, and icosahedron has been constructed.^8−13^ Such diversity provides rich opportunities for tailoring the PL properties.^2,14^

The QY can be enhanced by increasing the radiative decay (its rate constant *k*_r_) and/or decreasing the nonradiative decay (its rate constant *k*_*n*r_); note: QY = *k*_r_/(*k*_r_ + *k*_*n*r_). Doping is quite effective for enhancing the PL of NCs.^15−19^ Typically, introducing heterometal atoms into Au NCs does not change the parent structure.^15,16^ For example, Hirai et al. showed that doping Rh, Pt, and Ir into the Au_13_ superatom enhanced the QY from 11 to 46, 60, and 66%, respectively, whereas the QY of PdAu_12_ is comparable to that of Au_13_.^17^ The phenomenon was attributed to the larger energy gap of Rh@Au_12_, Pt@Au_12_, and Ir@Au_12_ than Pd@Au_12_ and Au_13_.^17^ Song et al. found that doping a gold atom into a cubic Cu_14_ cage gave a QY of 71%, much higher than that of the halide-centered Cu_14_ cage (QY ∼40%), which was attributed to the spin–orbit coupling effect from heavy gold atom.^20^

Without modifying the energy gap by size control or doping, suppression of nonradiative relaxation for Au NCs can be achieved by several strategies. For example, the body-centered cubic-structured Au_38_S_2_(SR)_20_ features locking gold atoms and the Au_4_S_4_ ring in between the units of the core, which gives a 15% QY at 900 nm emission.^21^ The nonradiative relaxation can be reduced by surface rigidification or the introduction of rigid ligands. Lee et al. obtained highly luminescent Au_22_(SG)_18_ by surface rigidification.^22^ A typical series of rigid ligands are the diphosphines, which show a decrease in structural flexibility with the chain length shortening, e.g., (Ph_2_)PCH_2_CH_2_P(Ph_2_) vs (Ph_2_)PCH_2_P(Ph_2_); hence, an enhancement of QY for the NCs.^16^ By changing the dimeric Au-SR motifs to trimeric and monomeric types while keeping the same kernel and ligand, Wu et al. found that the PLQY of face-centered cubic-structured Au_28_(SR)_20_ shows a 7-fold increment, which suggests that the staples can change the rigidity of the whole NC and thus suppress nonradiative relaxation.^23^ Ligands with high electron-donating capability can also enhance the QY by electron density transfer from ligands to the kernel.^24^ In recent work, Zhong et al. demonstrated that the QY of Au_10_ NCs can be drastically improved from <0.3 to 59.6% and even up to 90.3% by self-assembling two additional ligands via a layer-by-layer organization.^25^ It was found that the external noncovalent interactions such as hydrogen bonding and electrostatic forces play important roles in suppressing low-frequency vibrations of the kernel.^25^ Li et al. observed that the suppression of surface and kernel vibrations can be achieved by embedding Au_23_(SR)_16_^–^ NCs in polymer matrices and also by applying cryogenic conditions, which showed a large enhancement in QY from 3 to 40 and 70%, respectively.^26^

Motivated by these works, we aimed to evaluate the effect of the R group on reducing the nonradiative relaxation for medium-sized Au NCs protected by thiolate (–SR) because such sizes are rarely investigated in PL studies. To this end, we choose the Au_52_(SR)_32_ NC protected by aromatic ligands as a target because Au_52_ is a medium-sized system and the electronic and geometric structures are less affected by the change of substitution group on the –SR ligand and the energy gap of Au_52_ is in the NIR region^7^ but is not too small for efficient emission.

In this work, Au_52_(SR)_32_ NCs protected by different aromatic thiolate ligands, ***p*****-MBT** (R = –C_6_H_4_–CH_3_), **4-EBT** (R = –C_6_H_4_–CH_2_CH_3_), **IPBT** (R = –C_6_H_4_–CH(CH_3_)_2_), and **TBBT** (R = –C_6_H_4_-*t*Bu), are synthesized and their PL properties are investigated. Interestingly, the QY of Au_52_(SR)_32_ at room temperature in dichloromethane solution largely increases from 3.8 to 18.3% by reducing the number of methyl groups on the ligand’s *para*-position (**Au**_**52**_**(TBBT)**_**32**_ < **Au**_**52**_**(IPBT)**_**32**_ < **Au**_**52**_**(4-EBT)**_**32**_ < **Au**_**52**_**(*****p*****-MBT)**_**32**_). This behavior is also observed at the single-particle level by using total internal reflection fluorescence (TIRF) microscopy.^27^ The higher QY of **Au**_**52**_**(*****p*****-MBT)**_**32**_ than those of other Au_52_ counterparts (14.7% for **Au**_**52**_**(4-EBT)**_**32**_ and 11.1% for **Au**_**52**_**(IPBT)**_**32**_) is rare among the NIR emitters according to the energy gap law.^28^ We further reveal that the r.t. PL comprises phosphorescence and thermally activated delayed fluorescence. The effect of the ligand on the nonradiative relaxation is investigated by temperature-dependent PL and absorption measurements, facilitated by single-crystal X-ray diffraction (SCXRD) analysis. The observed R-dependent optical behavior suggests that the nonradiative relaxation can be suppressed by two routes: (1) restriction of vibrations and rotations of ligands by forming an ensemble through π···π interactions between the adjacent phenyl groups on the NC and (2) reducing the high-frequency vibrational quenching from the C–H bonds. Moreover, we find that Au_52_ with a less bulky substituent on the ligand endows a better capability for singlet oxygen (^1^O_2_) generation.

## Results and Discussions

### Synthesis of Four Au_52_(SR)_32_ NCs with Different R Groups

The details of the synthesis are described in the Supporting Information. Briefly, the synthesis of **Au**_**52**_**(*****p*****-MBT)**_**32**_ was performed under ambient conditions by the reduction of Au(I)-*p*-MBT polymers by using NaBH_4_. The crude product obtained after 1 h was further purified by preparative thin-layer chromatography (PTLC), which gave a yield of 7.5% (Au atom basis). A size-focusing step at an elevated temperature (70 °C) was required for the synthesis of **Au**_**52**_**(IPBT)**_**32**_. Initially, the reduction of Au(I)-IPBT polymers generated a polydisperse crude product, which was then subjected to excess IPBT etching at 70 °C for 5 h. Finally, PTLC was used to separate the target NC, which gave a yield of 2.7% (Au atom basis). The direct reduction of Au(I)-4-EBT polymers by NaBH_4_ could only produce a trace amount of **Au**_**52**_**(4-EBT)**_**32**_; therefore, a ligand-exchange-induced size/structure transformation (LEIST) method was carried out on **Au**_**52**_**(PET)**_**32**_ (a previously reported Au NC with a different kernel and ultraviolet–visible (UV–vis) absorption spectral profile, **PET = –**SCH_2_CH_2_Ph). First, **Au**_**52**_**(PET)**_**32**_ was prepared by the previous method.^29^ Then, **Au**_**52**_**(PET)**_**32**_ was subjected to etching with excess 4-EBT at 70 °C. The target NC was isolated by PTLC, with a yield of 20.4% (Au_52_(PET)_32_ basis). The synthesis of **Au**_**52**_**(TBBT)**_**32**_ followed a previous report^30^ with slight modifications.

### Photophysical Studies of Au_52_(SR)_32_ NCs at Ambient Conditions

**Figure 1:**
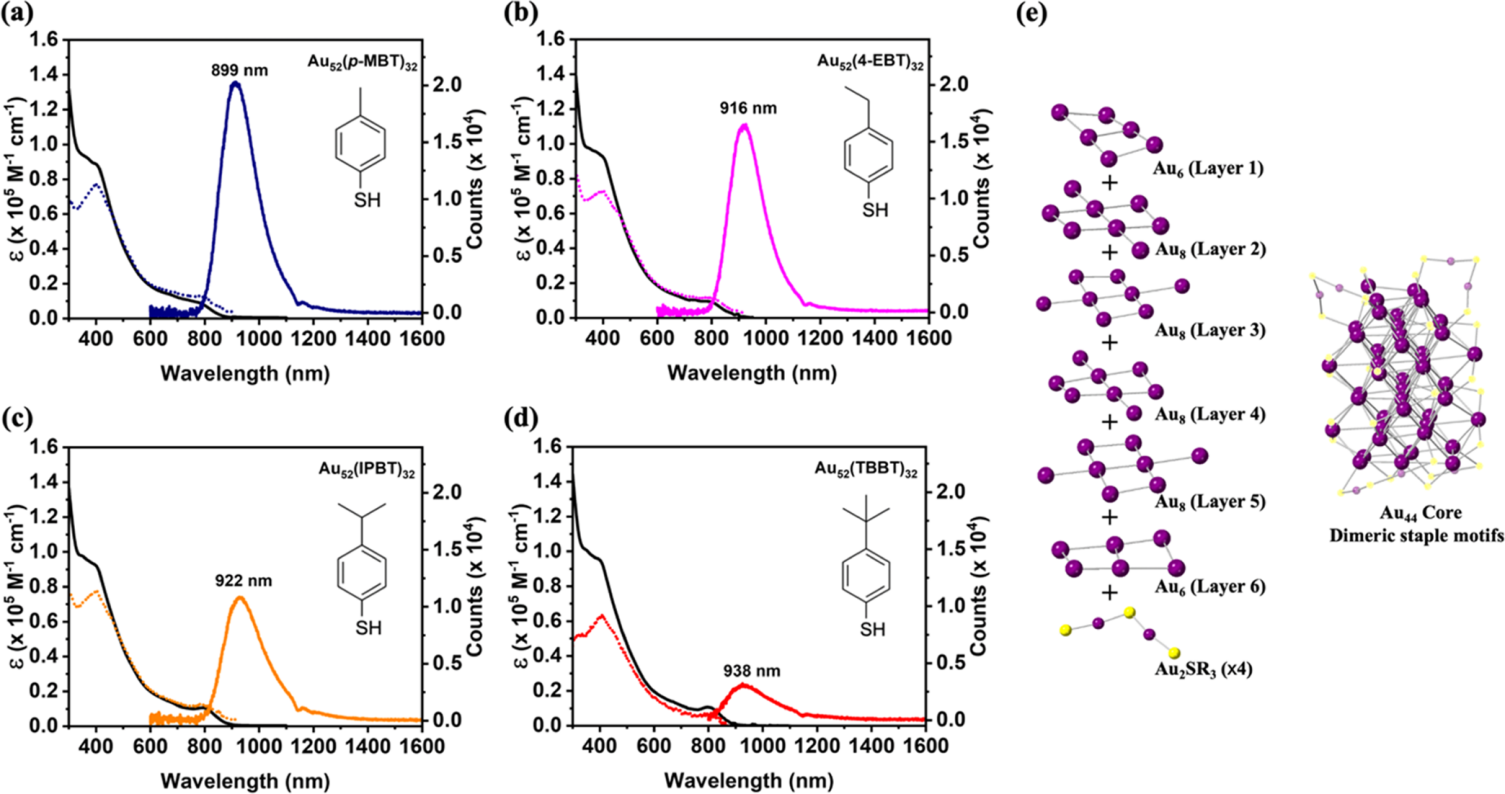
(a–d) Optical absorption spectra (black lines; molar absorption coefficients shown on the left *y*-axes), PL spectra (solid colored lines; photon numbers on the right *y*-axis), and normalized excitation spectra (dotted lines; right *y*-axis) of Au_52_(SR)_32_ with different ligands in DCM under ambient conditions. Slit width for PL and excitation measurements: 4 nm. (Note: the dip on the PL spectra at 1150 nm is due to the solvent reabsorption. The PL spectra were collected at 0.1 OD concentration at 470 nm excitation.) (e) Layer-by-layer arrangement of the kernel of Au_52_(SR)_32_ and the surface Au_2_(SR)_3_ staple motifs. Color code: purple = gold; yellow = sulfur; R groups are not shown for clarity.

The UV–vis absorption and PL properties of the four Au_52_(SR)_32_ NCs in dilute dichloromethane (DCM) solutions were investigated under ambient conditions (Figure 1). For all of the Au_52_ NCs (Figure 1a–d), the absorption band at ∼400 nm mainly consists of transitions from surface ligands and Au atoms in the staple motifs,^30^ while the long-wavelength peak at 800 nm (its onset at 890 nm, i.e., HOMO–LUMO gap, *E*_g_) is primarily due to the transitions in the kernels.^30,31^ The identical spectra indicate that these four NCs should have the same kernel and staple motifs as the previously reported layer-by-layer arrangement for the **Au**_**52**_**(TBBT)**_**32**_,^31^ which is shown in Figure 1e. The four Au_52_(SR)_32_ NCs exhibited a PL peak around 900–938 nm (1.32–1.39 eV) upon photoexcitation at 470 nm (2.64 eV). The agreement between absorption and excitation profiles (Figure 1a–d, **dashed lines**) indicates that the luminophores are the photoexcited Au_52_(SR)_32_ NCs, rather than any impurity, and that the PL originates from the *E*_g_ gap in the kernel. As the number of methyl groups in the *para*-position of the ligand’s phenyl group increases, the Stokes shift (SS) slightly increases from 0.172 to 0.199, 0.216, and 0.231 eV. This monotonic red-shift of the emission peak reflects a slight increment in the electron-donating ability of the substituent and can be ascribed to the ligand-induced change in the core’s electronic structure upon photoexcitation.^32^ Previous works on Au_25_(SR)_18_^–^ and Au_22_(C≡CR)_18_ found that the SS increase was associated with the increase of electron-donating ability of the ligands.^33−35^ Similar ligand-dependent trends on the SS are also found in our current work from the optical spectra of Au_52_(SR)_32_ after an exchange with different aromatic ligands: **Au**_**52**_**(*****p*****-MBT)**_**32-*****x***_**(4-MOBT)**_***x***_ (SS = 0.220 eV) and **Au**_**52**_**(*****p*****-MBT)**_**32–*****x***_**(4-FBT)**_***x***_ (SS = 0.161 eV) (Figures S1 and S2 in the SI), where, 4-MOBT stands for 4-methoxybenzenethiolate and 4-FBT for 4-fluorobenzenethiolate.

**Figure 2:**
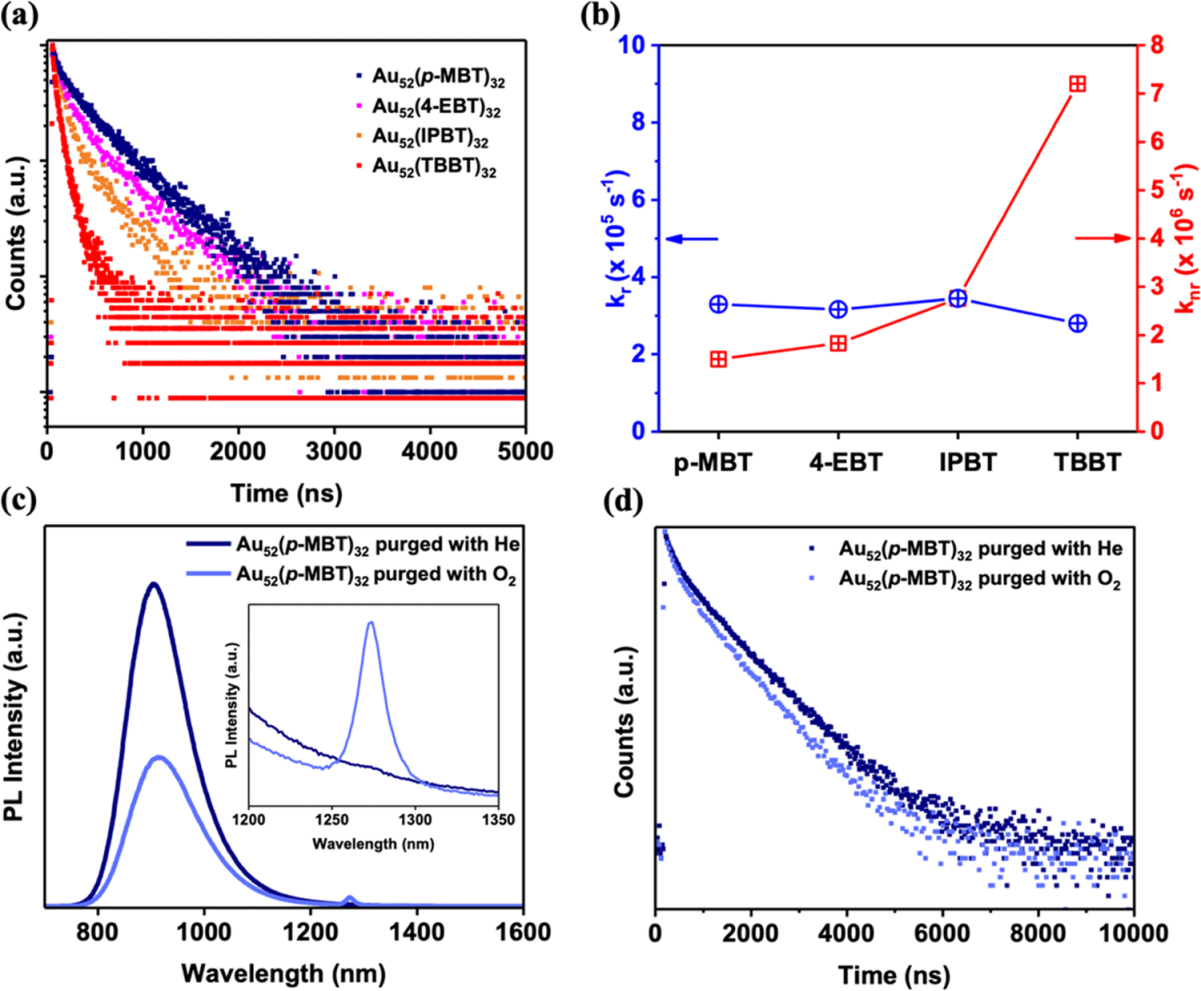
(a) PL decay profiles under ambient conditions in DCM. (b) Plot of radiative decay rate constant (blue symbol) and nonradiative decay rate constant (red symbol) of Au_52_(SR)_32_ protected by different ligands. (c) PL spectra of Au_52_(*p*-MBT)_32_ in tetrachloroethene under helium atmosphere and O_2_ atmosphere, respectively (excitation: 470 nm). (d) The corresponding PL decay profiles for helium and O_2_ atmospheres.

The PLQYs for **Au**_**52**_**(*****p*****-MBT)**_**32**_, **Au**_**52**_**(4-EBT)**_**32**_, **Au**_**52**_**(IPBT)**_**32**_, and **Au**_**52**_**(TBBT)**_**32**_ in diluted DCM solutions were determined by the absolute method (using an integrating sphere) under ambient conditions and are listed in Table 1. Notably, with fewer methyl groups on the *para*-position of the ligand, the QY of Au_52_ shows a monotonic increase and reaches 18.3% for Au_52_(*p*-MBT)_32_. The average PL lifetime, which was determined by the time-correlated single photon counting technique, also exhibits an R-dependent trend (Figure 2a) similar to that of QY. Given the small variation in the PL peak positions and the similar radiative rates (*k*_r_ ∼ 3 × 10^5^ s^–1^, see Table 1) among the four **Au**_**52**_**(SR)**_**32**_ NCs, we conclude that the electronic structures in both the ground state and the excited state should not be much affected by the R groups.^36^ Therefore, the prolonged PL lifetime with fewer substituents on the ligand is indicative of the suppression of the nonradiative relaxation for the excited NCs, which was observed previously in the studies of Ag_29_ NCs.^37−39^ The measured ln(*k*_*n*r_) values for Au_52_(SR)_32_ are between 14 and 16, which are close to the previous studies using nanosecond transient absorption spectroscopy.^7,40^ The comparison of their radiative rate constant (*k*_r_) and nonradiative rate constant (*k*_*n*r_) suggests that the enhancement in QY with fewer substituents is mainly associated with the suppression of nonradiative relaxation, rather than a faster radiative relaxation (Figure 2b). It should be noted that introducing fluorine at the *para*-position significantly decreases the QY, while the methoxy-substituted counterpart has a minor effect on the QY (Figures S1 and S2), suggesting that the electron-rich ligand favors a higher QY, which is similar to the [Au_25_(SR)_18_]^*q*^ NCs (*q* represents the charge).^23^ However, the QY is inversely proportional to the electron-donating capability of the ligands for the series **Au**_**52**_**(*****p*****-MBT)**_**32**_, **Au**_**52**_**(4-EBT)**_**32**_, **Au**_**52**_**(IPBT)**_**32**_, and **Au**_**52**_**(TBBT)**_**32**_, suggesting that the electronic effect at the *para*-position is counteracted by a much stronger effect of the ligand, which is identified to be the electron–vibration coupling (*vide infra*). The peak position and lifetime are barely changed under different solvent conditions and before/after ligand exchange, indicating that charge transfer^24,38^ is not a dominant factor for their PL properties (Figures S1–S3 in the SI).

**Table 1:** Photophysical Data for the Four Au_52_(SR)_32_ NCs in DCM Solution under Ambient Conditions^a^

solution phase	**Au**_**52**_**(*****p*****-MBT)**_**32**_	**Au**_**52**_**(4-EBT)**_**32**_	**Au**_**52**_**(IPBT)**_**32**_	**Au**_**52**_**(TBBT)**_**32**_
Φ_PL_ (%)	18.3	14.7	11.1	3.8
τ _ave_ (ns)	554	465	322	134
*k*_r_ (s^–1^)^b^	3.3 × 10^5^	3.2× 10^5^	3.5× 10^5^	2.8 × 10^5^
*k*_*n*r_ (s^–1^)^c^	1.5 × 10^6^	1.8× 10^6^	2.8× 10^6^	7.2 × 10^6^
*E*_g_ (eV)	1.4	1.4	1.4	1.4
PL (eV)	1.38	1.35	1.34	1.32

a The PL lifetimes are extracted from Figure 2a.

b Calculated by *k*_r_ = Φ_PL_·τ_ave_^–1^.

c Calculated by *k*_*n*r_ = (1 – Φ_PL_)·τ_ave_^–1^.

We further tested the PL sensitivity of Au_52_(SR)_32_ (dissolved in tetrachloroethene) to O_2_ (its ground state is a triplet, ^3^O_2_). After the solution was purged with O_2_, we found that the PL intensity was quenched to 51.4, 64.1, 65.8, and 84.6% of the initial PL intensity for **Au**_**52**_**(*****p*****-MBT)**_**32**_, **Au**_**52**_**(IPBT)**_**32**_, **and Au**_**52**_**(TBBT)**_**32**_, respectively (Figures 2c and S4). The O_2_-saturated solutions exhibited a distinctive emission peak from singlet oxygen (^1^O_2_, an excited state of ^3^O_2_) at 1274 nm and the average PL lifetime of Au_52_(SR)_32_ was slightly decreased (Figure 2d), suggesting that the triplet–singlet energy transfer occurred between Au_52_(SR)_32_ and oxygen. The as-confirmed triplet state population of Au_52_(SR)_32_ implies that its emission can be phosphorescence and/or thermally activated delayed fluorescence (TADF)^41−43^ because both types of PL are associated with the population in the triplet excited state (T_1_). Since the variation in the ligand series is small (i.e., merely the number of CH_3_ groups), the emission mechanism should be the same for the four Au_52_(SR)_32_ NCs. In the tetrachloroethene solution of **Au**_**52**_**(*****p*****-MBT)**_**32**_, its average PL lifetime (τ_ave_) is 700 ns (component τ_1_ = 187.0 ns (19.8%) and τ_2_ = 827.8 ns (80.2%)) under O_2_ and increases to 807 ns (component τ_1_ = 196.8 ns (14.8%) and τ_2_ = 915.4 ns (85.2%)) under the helium atmosphere. The longer lifetime in tetrachloroethene (τ_ave_= 700 ns) than that in DCM (τ_ave_ = 554 ns) indicates a weaker interaction of solvent dipoles with the Au_52_ excited-state dipole due to the less polar nature of tetrachloroethene and hence a longer PL lifetime.

When the four Au_52_(SR)_32_ NCs were each embedded in a poly(methyl methacrylate) (PMMA) film, the QYs (Table 2) for **Au**_**52**_**(*****p*****-MBT)**_**32**_, **Au**_**52**_**(4-EBT)**_**32**_, **Au**_**52**_**(IPBT)**_**32**_, and **Au**_**52**_**(TBBT)**_**32**_ were largely increased to 1.85, 3.2, 2.4, and 5.8 times the solution QY, respectively (comparing Tables 1 and 2). Meanwhile, the average PL lifetimes were increased to 808, 768, 423, and 409 ns, respectively (Figure S5). These changes result from the suppression of ligand-induced nonradiative relaxation, which is reflected in the large decrease in *k*_*n*r_ (Table 2) compared to the solution state.

**Table 2:** Photophysical Data for Au_52_(SR)_32_ NCs Embedded in PMMA Films

polymer film	**Au**_**52**_**(*****p*****-MBT)**_**32**_	**Au**_**52**_**(4-EBT)**_**32**_	**Au**_**52**_**(IPBT)**_**32**_	**Au**_**52**_**(TBBT)**_**32**_
Φ_PL_ (%)	33.9	32.2	26.6	22.1
τ _ave_ (ns)	808	768	423	409
*k*_r_ (s^–1^)	4.2 × 10^5^	4.2 × 10^5^	2.5 × 10^5^	4.5 × 10^5^
*k*_*n*r_ (s^–1^)	8.2 × 10^5^	8.8 × 10^5^	1.7 × 10^6^	1.6 × 10^6^

### Insights into the PL Mechanism of Au_52_(SR)_32_

**Figure 3:**
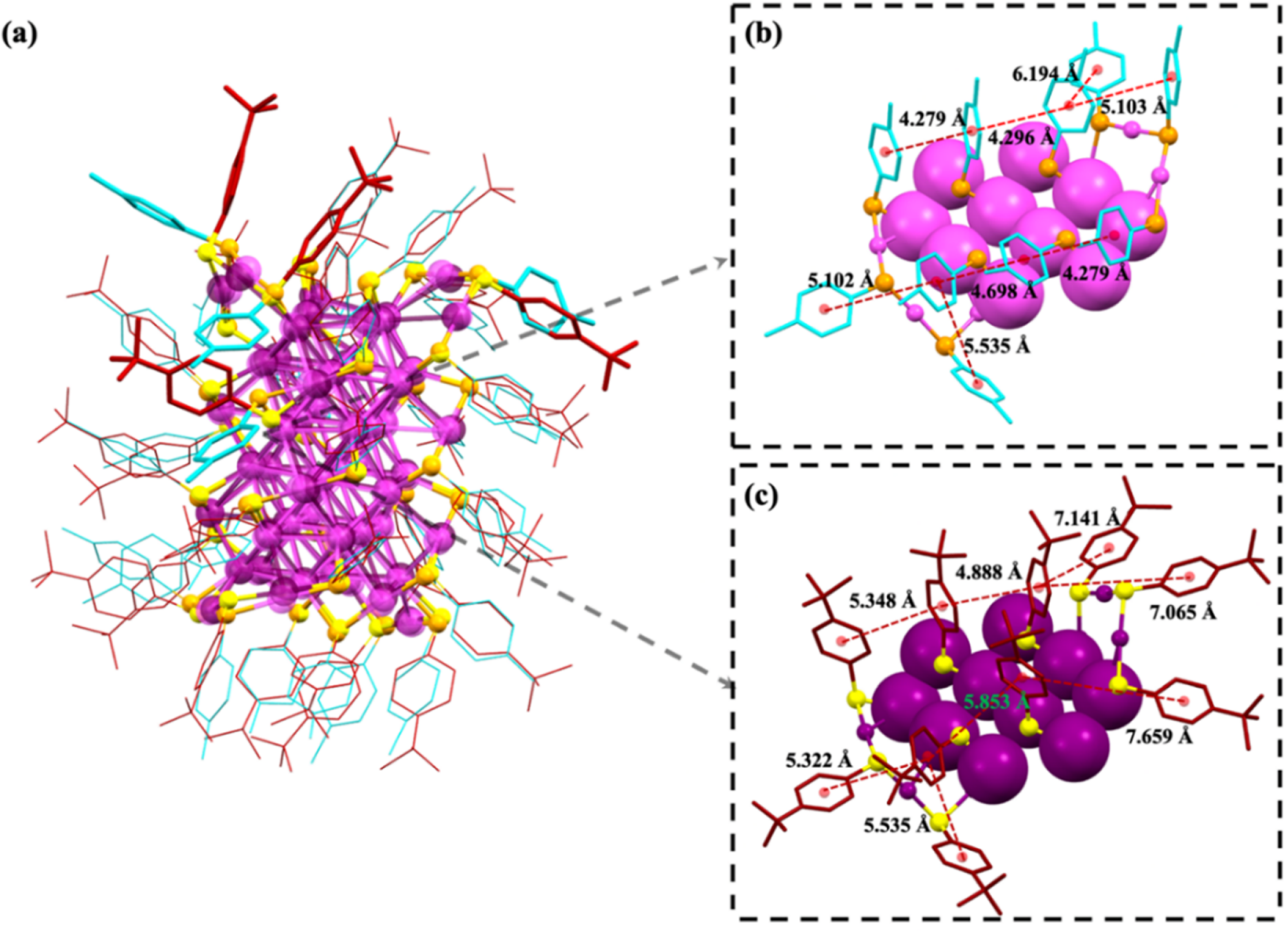
(a) Overlay of **Au**_**52**_**(*****p*****-MBT)**_**32**_ and **Au**_**52**_**(TBBT)**_**32**_ SCXRD structures captures the orientation differences of carbon tails. The exceptional differences in four ligands for the two Au_52_ are shown in bold style. The side views of **Au**_**52**_**(*****p*****-MBT)**_**32**_ (b) and **Au**_**52**_**(TBBT)**_**32**_ (c). Color code: purple and magenta = gold; yellow and orange = sulfur; red = carbon on TBBT; light blue = carbon on *p*-MBT.

The four Au_52_ NCs feature the same core and staple motifs, as indicated by the similar UV–vis absorption profiles and also confirmed by the determined Au_52_(*p*-MBT)_32_ X-ray structure (*vide infra*). We rationalize that the enhancement in QY for the *p*-MBT-protected Au_52_ NC may originate from two mechanisms: (1) the nonradiative relaxation via the coupling between the excited electron and the high-frequency C–H vibrations (∼3000 cm^–1^)^44,45^ should be suppressed in Au_52_(*p*-MBT)_32_ compared to other Au_52_(SR)_32_ NCs with more −CH_3_ groups; and (2) the orientation of phenyl rings on Au_52_ with less bulky ligands (i.e., fewer methyl groups at the *para*-position) may lead to stronger interligand π···π stacking interaction on the core, which facilitates the suppression of intramolecular vibrational and rotational motions.^46,47^ For mechanism 1, the photoexcited electron can relax via a multiphonon process (*E*_g_ ∼ 1.38 eV requires nearly four phonons of C–H vibrations); the fewer −CH_3_ groups in Au_52_(*p*-MBT)_32_ reduces the amplitude of the multiphonon decay, hence, a higher QY. For mechanism 2, the relatively large {100} facets of Au_52_ may result in stronger intracluster ligand π···π interactions, which suppress nonradiative relaxation. To confirm the rationale, we grew single crystals of Au_52_(*p*-MBT)_32_ by the vapor-diffusion of acetonitrile into a concentrated toluene solution of Au_52_(*p*-MBT)_32_. The SCXRD analysis revealed that the core structure of Au_52_(*p*-MBT)_32_ was the same as that of the previously reported Au_52_(TBBT)_32_ (see the overlaid cores in Figure 3a and also Figure S6 for a comparison of bond lengths). Despite the same core structure, distinct differences in the packing of surface ligands can be seen; specifically, the average distance between adjacent benzene rings is shorter in **Au**_**52**_**(*****p*****-MBT)**_**32**_ than in **Au**_**52**_**(TBBT)**_**32**_ (Figure 3b,c), i.e., 4.794 vs 5.481 Å, hence, more strongly restricting the ligand motions in **Au**_**52**_**(*****p*****-MBT)**_**32**_. Therefore, the different orientations of the ligands on Au_52_ NCs and ligand spacings should be responsible for their QY difference^48^ in addition to the different amplitudes of multiphonon nonradiative decay. Meanwhile, the trends of PL intensity and lifetime are consistent in the DCM solution and in the solid form (PMMA films), which supports the existence of the interligand π···π stacking interactions in the solution.

### Temperature-Dependent PL of Au_52_(*p*-MBT)_32_

**Figure 4:**
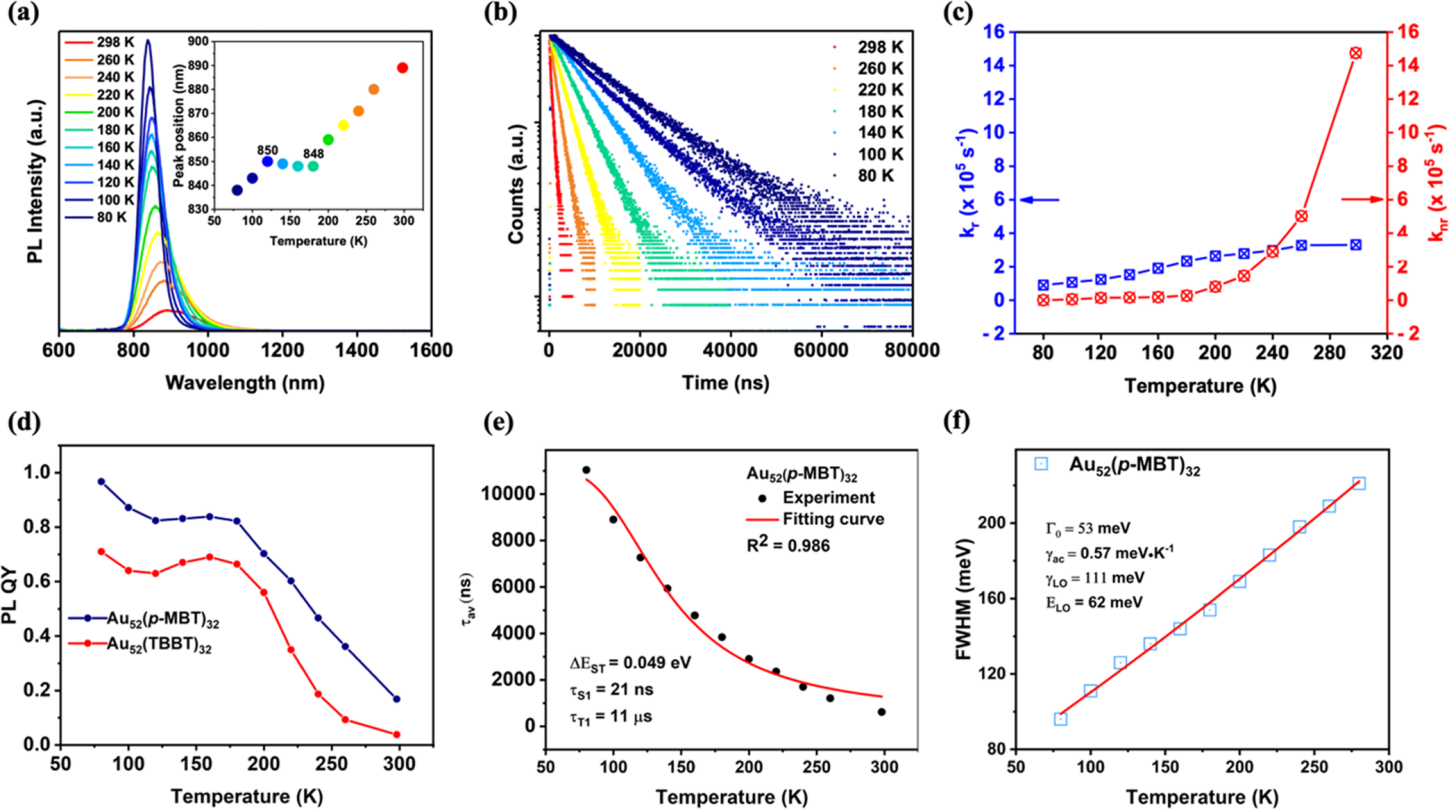
(a) Temperature-dependent PL spectra of Au_52_(*p*-MBT)_32_ in 2-methyl-THF under a He atmosphere. (b) PL decay profiles at selected temperatures. (c) Plot of radiative decay rate constants (blue symbols) and nonradiative decay rate constants (red symbols) from 80 to 298 K. (d) Temperature-dependent PL quantum yields of Au_52_(*p*-MBT vs TBBT, similar for other ligands) in solution (note: “glass” formation at low temperatures). (e) Temperature-dependent emission lifetime of Au_52_(*p*-MBT)_32_ and the fitting of data by eq 2. (f) fwhm of the PL spectra as a function of temperature for Au_52_(*p*-MBT)_32_. The red line is the fitting result shown in eq 3.

To understand the PL nature, temperature-dependent photophysical investigations on PL spectra and lifetimes were further carried out from 298 to 80 K (see **Au**_**52**_**(*****p*****-MBT)**_**32**_ in Figure 4a,b and the other three NCs in Figures S7–S9). Of note, in order to form clear “glass” at cryogenic temperatures for PL measurements, 2-methyl-THF was used to dissolve the NCs at room temperature, followed by temperature-dependent measurements. The temperature-dependent *k*_r_ and *k*_*n*r_ values for **Au**_**52**_**(*****p*****-MBT)**_**32**_ are extracted and plotted in Figure 4c. Upon reduction in temperature, the PL peak became sharper due to the weakening of electron–vibration coupling. Note: the lattice expansion contribution is minor in the NCs.^49^ The enhancement of PL with decreasing temperature is due to the suppression of nonradiative relaxation, which is indicated by the prolonged lifetime and the drop in the *k*_*n*r_ value (Figure 4b,c). Interestingly, the PL peak position exhibits an anomaly between 200 and 120 K (nearly a plateau, Figure 4a, inset) rather than the normal blue-shift with decreasing temperature. However, below 120 K, the monotonic blue-shift of the peak position reappears. Similarly, the PLQY first increases and reaches 82% at ∼180 K but then decreases over 160–120 K and finally increases again (Figure 4d, similar for other ligand cases). These abnormal trends were observed in all four Au_52_(SR)_32_ NCs and indicate that TADF is involved,^42^ rather than sole phosphorescence, because, if the phosphorescence is the sole emission, it would monotonically increase and sharpen with decreasing temperature and would also monotonically blue-shift, rather than exhibiting the observed anomalies.

The TADF process^50,51^ is governed by the reverse intersystem crossing (RISC) rate, *k*_RISC_, and can be estimated by the Arrhenius equation^43,52^
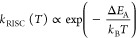
1where *k*_B_ is the Boltzmann constant, *T* is the temperature, and Δ*E*_A_ is the activation energy to reach the crossing seam between the S_1_ and T_1_ potential energy surfaces. Solvent reorganization energy is assumed to be insignificant for Δ*E*_A_, so the Δ*E*_A_ value can be estimated by the singlet–triplet energy separation Δ*E*_S-T_.^53^ Below 200 K, the RISC process started to be suppressed in the Au_52_(SR)_32_ NCs, and the emission mainly arises from the triplet state (i.e., phosphorescence). As expected for the TADF mechanism, the material should exhibit a small singlet–triplet separation Δ*E*_S-T_, which is confirmed by fitting the temperature-dependent lifetime with the Boltzmann-type model^54^ (eq 2) (Figures 4e and S10). The extracted parameters are listed in Table 3. As shown in Table 3, the fitted Δ*E*_S-T_ are 49, 59, 59, and 83 meV for **Au**_**52**_**(*****p*****-MBT)**_**32**_, **Au**_**52**_**(4-EBT)**_**32**_, **Au**_**52**_**(IPBT)**_**32**_, and **Au**_**52**_**(TBBT)**_32_, respectively. These values are lower in energy than the PL spectral blue-shift (from 899 to 838 nm, or ∼100 meV blue-shift) during the temperature decrease, suggesting that a change in the emission proportion (TADF/phosphorescence) occurred in a narrow temperature range. The temperature-dependent PL line width (full-width at half-maximum, fwhm) of Au_52_(*p*-MBT)_32_ is further extracted and plotted in Figure 4f, which presents a nearly linear relationship to the temperature (from 80 to 280 K), suggesting a weak electron–phonon interaction represented by eq 3,^55^ in which Γ_0_ is the PL line width at 0 K, γ_ac_ and γ_LO_ are the coupling coefficients of the excited electron with acoustic phonon (ac) and longitudinal optical (LO) phonon, respectively, and *E*_LO_ represents the average energy of the LO phonon. Data fitting to eq 3 gives the fitted parameters (Figure 4f, inset). It is worth noting that the coupling of optical phonons with the electron in Au_52_(*p*-MBT)_32_ (γ_LO_ = 111 meV) is significantly less than that in the classic Au_25_(PET)_18_^–^ NC (γ_LO_ = 423 meV), which explains the higher QY of Au_52_(*p*-MBT)_32_ than that of Au_25_(SR)_18_^–^ (QY ∼ 1%).^54^
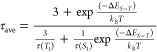
2
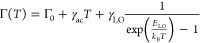
3

**Table 3:** Results from Fitting of Temperature-Dependent PL Lifetimes for the Four Au_52_(SR)_32_ NCs

NCs	**Au**_**52**_**(*****p*****-MBT)**_**32**_	**Au**_**52**_**(4-EBT)**_**32**_	**Au**_**52**_**(IPBT)**_**32**_	**Au**_**52**_**(TBBT)**_**32**_
Δ*E*_S-T_, meV	49	59	59	83
intrinsic τ(S_1_), ns	21	97	78	24
intrinsic τ(T_1_), μs	11	9.5	8.9	7
QY at 180 K, Φ_PL_	82%	77%	70%	66%

### Temperature-Dependent Optical Absorption of Au_52_ NCs

**Figure 5:**
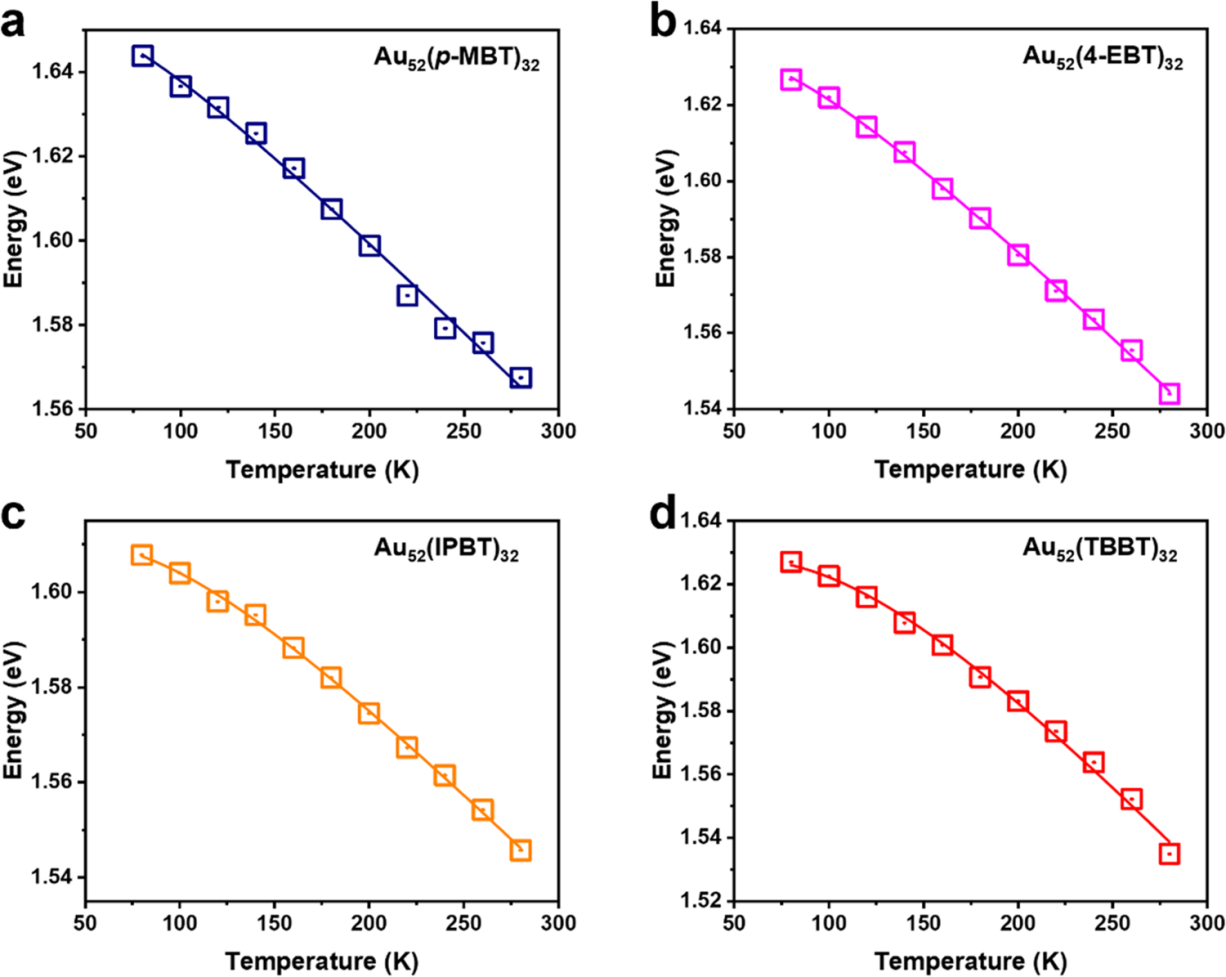
Temperature-dependent trends and fitting results of the absorption maxima for the four Au_52_(SR)_32_ NCs (indicated in panels (a–d)).

To further understand the electron–vibration coupling in Au_52_ NCs, we further carried out temperature-dependent optical absorption measurements on the four Au_52_ NCs from r.t. down to 80 K. As shown in Figure S11, the absorption spectra of all four Au_52_ NCs exhibit a distinct blue-shift for the HOMO–LUMO transition (peak ∼1.55 eV at room temperature) and an increase in the oscillator strength with decreasing temperature. Such trends are caused by the suppression of phonon populations at low temperatures and are consistent with the previous observations in Au_25_ and Au_38_.^49,56^ Here, we apply a modified Bose–Einstein single oscillator model (eq 4) that was developed by O’Donnell and Chen to describe the absorption peak dependence on temperature^57^
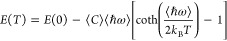
4In this model, all of the vibrational modes that contribute to the electron–phonon coupling of the specific electronic transition are simplified as a single oscillator,^58^ i.e., ⟨ℏω⟩ as the average energy of all vibrational modes, ⟨C⟩ represents the electron–phonon coupling strength, and *E*(0) is the electronic transition gap at 0 K. Since the emissions of the four Au_52_ NCs follow Kasha’s rule (i.e., PL from the lowest excited state only), we focus on the temperature-dependent evolution of *E*_g_, and the absorption maxima at ∼1.6 eV is used as the value of *E*_g_. The temperature-dependent *E*_g_ values of the four Au_52_ NCs are extracted from Figure S11 and plotted in Figure 5, in which the solid lines represent the fitting by eq 4, and the extracted parameters are given in Table 4.

**Table 4:** Results from Fitting of Temperature-Dependent HOMO–LUMO Transition in the Four Au_52_(SR)_32_ NCs (Note: 1 meV = 8 cm^–1^)

	*E*(0)/eV	⟨C⟩	⟨ℏω⟩/meV
Au_52_(*p*-MBT)_32_	1.653	2.6	16
Au_52_(4-EBT)_32_	1.634	2.9	19
Au_52_(IPBT)_32_	1.611	3.4	26
Au_52_(TBBT)_32_	1.628	3.8	32

Interestingly, the extracted average energy of vibrational modes that are coupled to the HOMO–LUMO transition presents a gradual decrease as the number of −CH_3_ groups at the *para*-position of the ligand decreases. Such a phenomenon indicates lower energy vibrational modes coupled to the HOMO–LUMO transition when the number of methyl groups decreases. The 260 cm^–1^ (32 meV) vibrational mode in Au_52_(TBBT)_32_ suggests that the Au_2_(TBBT)_3_ staple motifs are largely involved in the nonradiative energy dissipation, while the 130 cm^–1^ (16 meV) vibrational mode in Au_52_(*p*-MBT)_32_ indicates that the core vibrations (typically <200 cm^–1^) are the main contributor to the nonradiative relaxation. Therefore, the gradual decrease of the average energy of vibrational modes from Au_52_(TBBT)_32_ to Au_52_(*p*-MBT)_32_, as well as the decreasing coupling strength ⟨C⟩, explains that the suppression of surface vibrations is the main reason for the decrease in nonradiative relaxation. With significantly suppressed surface vibrations, Au_52_(*p*-MBT)_32_ gives rise to the highest QY among the series and is also the highest QY reported for Au_*n*_(SR)_*m*_ in the NIR range, albeit near unity QY in the visible range^24^ has been reported by Zhong et al.

### Singlet Oxygen Generation Capability and Single-Cluster PL

**Figure 6:**
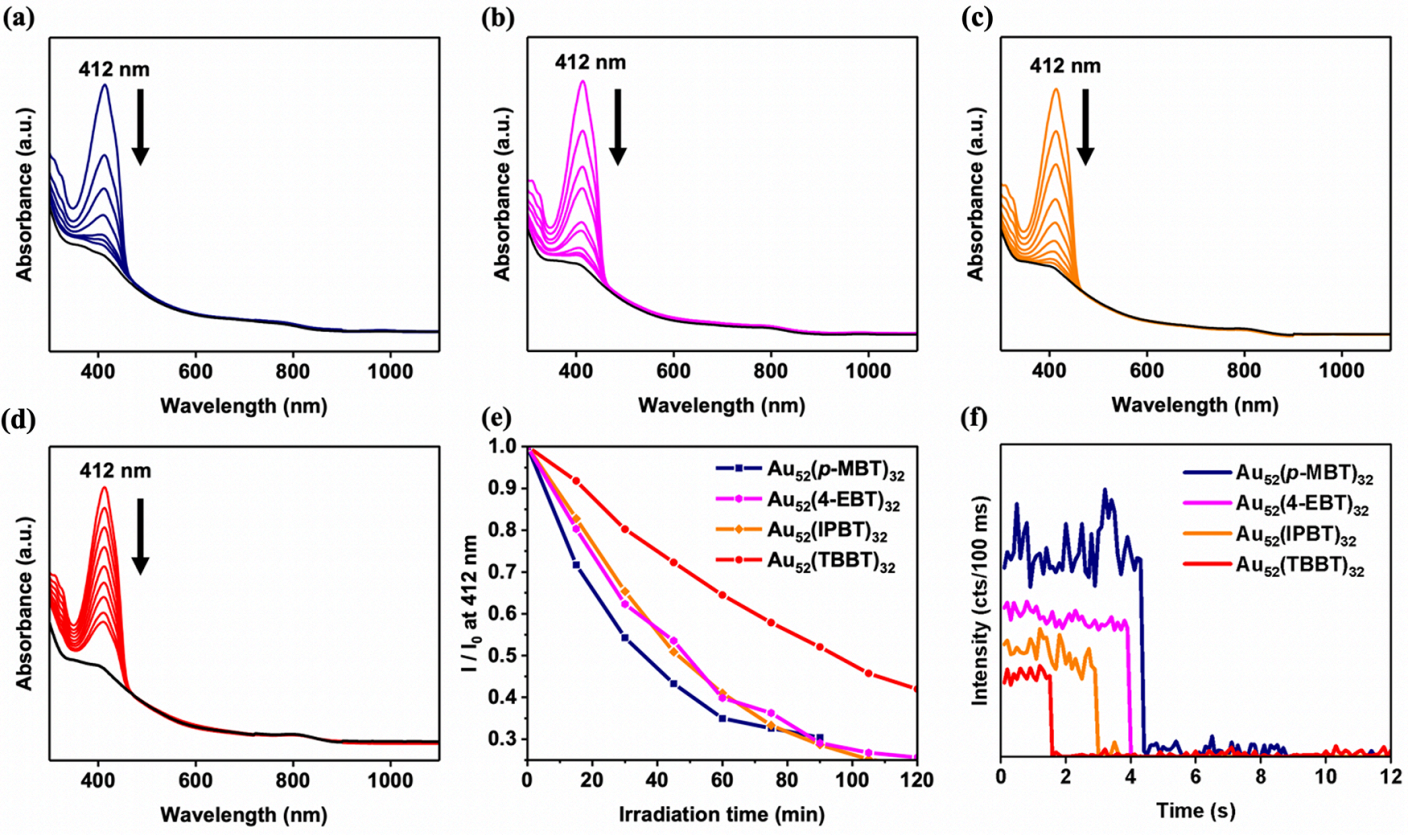
Absorption spectra of DPBF (60 μg/mL) in DMF solutions of Au_52_(SR)_32_ NCs (concentration is adjusted to the same optical density at 470 nm): (a) **Au**_**52**_**(*****p*****-MBT)**_**32**_, (b) **Au**_**52**_**(4-EBT)**_**32**_, (c) **Au**_**52**_**(IPBT)**_**32**_, and (d) **Au**_**52**_**(TBBT)**_**32**_. (e) Plot of the 412 nm peak intensity change (%) against the irradiation time, where *I*_0_ is the initial intensity. (f) Time trajectory study of the four **Au**_**52**_**(SR)**_**32**_ NCs using TIRF.

1,3-Diphenylisobenzofuran (DPBF) is a common probe used for sensing ^1^O_2_ in an organic solvent, which absorbs strongly at 412 nm. The photosensitized ^1^O_2_ readily reacts with DPBF to form 1,2-dibenzoylbenzene (DBB) and bleaches the absorption band at 412 nm (Figure 6a–d).^59,60^ The time-dependence of the absorption intensity change at 412 nm for the oxygen-saturated DMF solution containing an equal concentration of various Au_52_(SR)_32_ (all at 8 μM) and DPBF (10 μM) under irradiation (500 nm) are plotted in Figure 6e. To evaluate the net effect from Au_52_(SR)_32_, the blank solution in the absence of Au_52_(SR)_32_ (Figure S12) was subtracted from the plots in the presence of Au_52_(SR)_32_. For all four Au_52_(SR)_32_ NCs, the 412 nm band of DPBF gradually decreased over 120 min. Of note, the absorption band at 800 nm for Au_52_(SR)_32_ NCs was not bleached upon irradiation for 120 min, indicating that their structures remained intact during the process. The observed trend for the ^1^O_2_ generation rate in Figure 6e is consistent with the QY trend of the four Au_52_(SR)_32_ NCs, suggesting that the Au_52_ protected by less steric ligand has a higher efficiency in generating ^1^O_2_ because a less bulky substituent at the *para*-position allows more efficient interaction between the metal core and ^3^O_2_ in the solvent.

The four Au_52_ NCs were further studied at the single-particle level using total internal reflection fluorescence (TIRF) microscopy^27^ under a 488 nm continuous laser irradiation in air. The order of the single NC’s PL intensity (Figure 6f) is the same as the ensemble (i.e., solution) PL order, indicating that the QYs of Au_52_(SR)_32_ from ensemble measurements do not involve any aggregation-induced emission (AIE) effect.^61^ In addition, the emission was relatively stable on the timescale of the image capture (100’s of ms), which is advantageous for their applications in imaging.

## Conclusions

In summary, we have investigated the effect of the *para*-structure of the –SR ligand on the PL performance of Au_52_(SR)_32_ NCs and offered mechanistic insights. By reducing the *para*-bulkiness of the ligands, the nonradiative pathway can be effectively suppressed, thereby enhancing the PLQY. Among them, Au_52_(*p*-MBT)_32_ shows a PLQY of 18.3% in a non-degassed DCM solvent at room temperature, which is rare for NIR emitters. The present result reveals two factors for suppressing the nonradiative pathways: (1) reduction of the ligand’s motions via π···π stacking interaction between the adjacent phenyl rings and (2) suppression of high-frequency vibrations from the C–H oscillators. This work has also elucidated the PL nature in Au_52_(SR)_32_, which involves phosphorescence and TADF; the latter is manifested in the abnormal spectral shift and a zigzag change in PL intensity with decreasing temperature. Finally, the ligand effect on the ^1^O_2_ generation efficiency increases with the decrease of the *para*-bulkiness for the ligands. The obtained mechanisms and highly NIR-luminescent nanoclusters will promote the design of NIR emitters and the development of their applications in photonics, bioimaging, solar energy conversion, and many other fields.

## Supporting Information Available

Experimental part includes the synthesis, PL analysis, cryogenic optical absorption, PL, and TIRF analysis; PL spectra of Au_52_(*p*-MBT)_32_ in different solvents (PL intensities: Tol.∼DCM > THF > DMF); blank DPBF solution in DMF in the absence of gold nanoclusters under irradiation with λ= 500 nm; cryogenic optical absorption spectra at different temperature; plots of emission lifetime collected at 470 nm excitation against temperature for the cases of 4-EBT, IPBT and TBBT; sample and crystal data for Au_52_(S-*p*-MeC_6_H_4_)_32_; X-ray structure (PDF)

## Accession Codes

CCDC 2295328 contains the supplementary crystallographic data for this paper. These data can be obtained free of charge via www.ccdc.cam.ac.uk/data_request/cif, or by emailing data_request@ccdc.cam.ac.uk, or by contacting The Cambridge Crystallographic Data Centre, 12 Union Road, Cambridge CB2 1EZ, UK; fax: +44 1223 336033.
