# Supplementary material for: Tailoring Carbon Tails of Ligands on Au52(SR)32 Nanoclusters Enhances the Near-Infrared Photoluminescence Quantum Yield from 3.8 to 18.3%
Source: J Am Chem Soc. 2023 Nov 20;145(48):26328–38. doi: 10.1021/jacs.3c09846 (PMC10704554; doi:10.1021/jacs.3c09846)
Supplement: Supplementary file 1 — ja3c09846_si_001.pdf [file ja3c09846_si_001.pdf]

Supporting Information:

Tailoring Carbon Tails of Ligands on $Au_{52}(SR)_{32}$ Nanoclusters Enhances Near-Infrared Photoluminescence Quantum Yield from 3.8% to 18.3%

Yitong Wang[†], Zhongyu Liu[†], Abhrojyoti Mazumder[†], Christopher G Gianopoulos[‡], Kristin Kirschbaum[‡], Linda A Peteanu[†], Rongchao Jin[†*]

[†]Department of Chemistry, Carnegie Mellon University, Pittsburgh, Pennsylvania 15213, United States

[‡]Department of Chemistry and Biochemistry, University of Toledo, Toledo, Ohio 43606, United States

*To whom correspondence should be addressed: rongchao@andrew.cmu.edu

Experimental:

1. Materials and reagents

Tetrachloroauric (III) acid ($HAuCl_4 \cdot 3H_2O$, 99.999% metal basis, Aldrich), tetraoctylammonium bromide (TOAB, ≥98%, Fluka), 4-methylbenzenethiol (*p*-MBT, C_7H_7SH, 98%, Aldrich), 4-tert-butylbenzenethiol (TBBT, $C_{10}H_{13}SH$, >97.0%, TCI), 4-ethylbenzenethiol (4-EBT, 97%, TCI America), 4-isopropylbenzenethiol (IPBT, TCI, ≥99.8 %), 4-fluorobenzenethiol (4-FBT, 98%, Aldrich), 4-methoxybenzenethiol (4-MOBT, 97%, Aldrich), benzenethiol (HSPh, 97%, Aldrich), sodium borohydride ($NaBH_4$, Aldrich), and 1,3-diphenylisobenzofuran (DPBF, 95%, Aldrich).

Solvents: methanol (HPLC grade, ≥99.9%, Aldrich), tetrahydrofuran (THF, HPLC grade, ≥99.5%), dichloromethane (DCM, ACS reagent, ≥99.5%, Aldrich), toluene (HPLC grade, ≥99.9%, Aldrich), hexane (ACS grade, ≥99.9%, Aldrich), 2-methyltetrahydrofuran (anhydrous, inhibitor-free, ≥99%, Aldrich).

All chemicals were used without further purification. Nanopure water was prepared with a Barnstead NANOpure Diamond system. Thin-layer chromatography (TLC) plates were from iChromatography (silica gel, 250 μm).

2. Synthesis

The syntheses of **$Au_{28}(TBBT)_{20}$**, **$Au_{36}(TBBT)_{24}$**, **$Au_{44}(TBBT)_{28}$**, and **$Au_{52}(TBBT)_{32}$** were carried out under ambient conditions, followed by separation with TLC plates (developing solvents: 2:5 (v/v) DCM: n-hexane). Further purifications were also performed on TLC plates.

Typically, a 50 ml round-bottom flask equipped with a magnetic stir bar was charged with $HAuCl_4 \cdot 3H_2O$ (78.74 mg, 0.2 mmol). 15 ml of THF containing TOAB (136.7 mg, 0.25 mmol) was added to the flask under rapid stirring (550 rpm). The solution turned to deep orange in 20 minutes. Then, 133 μL TBBT (0.8 mmol) was added to the solution. After 30 minutes of stirring, the solution changed to light yellow, indicating the formation of $Au_{10}(TBBT)_{10}$ complex. A freshly prepared $NaBH_4$ solution (38 mg, 1 mmol) was rapidly added to the reaction mixture. Upon mixing, the solution immediately turned black. The reaction was allowed to proceed for one hour. Then, the aqueous phase was removed by a glass pipet, and the organic phase was concentrated to ~3 mL under reduced pressure. The organic phase was washed with excess methanol (about 12 ml) five times to remove excess thiol. In the second step, 200 μL TBBT was added to the dark residue that was dissolved in 2 mL toluene. The solution was heated to 50 ºC and maintained at the temperature under an air atmosphere with gentle stirring (~60 rpm) for 16 hours. Then,

the product was separated from the thiol/toluene solution by washing with excess methanol. The dark residue was extracted by a minimum amount of DCM and then purified by TLC plates.

Au_{52}(*p*-MBT)$_{32}$. A vail was charged with $HAuCl_4{\cdot}3H_2O$ (78.74 mg, 0.2 mmol). TOAB (136.7 mg, 0.25 mmol) was put in THF (15 ml) and added to the vail. Under stirring, the mixture becomes deep orange in 20 minutes. Then, *p*-MBT (146 mg, 1 mmol) was added to the vial. The solution was kept stirring for 60 minutes until the color of the mixture turned to light yellow. Subsequently, $NaBH_4$ (26.6 mg, 0.7 mmol, dissolved in 5 mL of ice-cold nanopure water) was added to the vail all at once. After 60 minutes of reaction, the aqueous phase was removed, and the black organic phase was dried by rotary evaporation. The precipitates were washed with methanol five times. The size-mixed Au_x(*p*-MBT)$_y$ NCs were extracted from the precipitates with a small amount of DCM and dried. Further purification was conducted by PTLC. The PTLC was developed with a mixture of DCM and n-hexane (2:3 (v/v)). Block-like Au_{52}(*p*-MBT)$_{32}$ single crystals were obtained by vapor diffusion of CH_3CN into a toluene solution of the nanoclusters at 4 °C. In another method, needle-like Au_{52}(*p*-MBT)$_{32}$ single crystal were obtained by vapor diffusion of methanol into a 1,2,4-trichlorobenzene solution of the nanoclusters.

Au_{52}(4-EBT)$_{32}$. First, $Au_{52}(PET)_{32}$ was synthesized and separated by PTLC according to the previous work[s1]. In the second step, ~3 mg of $Au_{52}(PET)_{32}$ was dissolved in 2 mL toluene and 200 μL 4-EBT. The mixture was heated at 70 °C for 5 hours. The crude products were washed with excess methanol and dissolved in 2 mL of DCM. Then, the target product was isolated from the crude product by PTLC (DCM and n-hexane (1:2 (v/v)).

$Au_{52}(IPBT)_{32}$. In the first step, $HAuCl_4{\cdot}3H_2O$ (100 mg, 0.254 mmol) was dissolved in 15 mL of THF. TOAB (164 mg, 0.3 mmol) was added to the solution. The mixture became deep orange in 20 minutes. Then, 145 μL IPBT was added to the vail. After the color of the mixture turned to light yellow (in ~30 minutes), a freshly prepared $NaBH_4$ solution (38 mg, 1 mmol, dissolved in 5 mL of ice-cold nanopure water) was added all at once. After stirring overnight, the reaction was stopped, and the solution was evaporated to dryness. The brown residue was washed with excess methanol and dissolved in a minimum amount of toluene (~2 mL). Next, 400 μL IPBT was added to the above solution and heated to 70 °C for 5 hours. After that, methanol was added to the reaction mixture to precipitate the product, followed by centrifugation at 10,000 rpm. The solid product was washed with excess methanol five times to remove excess thiol. Finally, the residue was dissolved in a small amount of DCM (~1 ml) and purified by PTLC using a mixture of DCM and n-hexane (2:5) as a developing solvent.

Au_{52}(*p*-MBT)$_{32-x}$(4-FBT)$_x$. First, Au_{52}(*p*-MBT)$_{32}$ was synthesized by the above method. Next, ~1.5 mg of Au_{52}(*p*-MBT)$_{32}$ was heated in the presence of 4-FBT (200 μL added) at 60 °C for 5 hours. The resulting mixture was washed with excess methanol and further purified by PTLC using a mixture of DCM and n-hexane (2:1) as the developing solvent.

Au_{52}(*p*-MBT)$_{32-x}$(4MOBT)$_x$. First, Au_{52}(*p*-MBT)$_{32}$ was synthesized by the above method. Next, ~1.5 mg of Au_{52}(*p*-MBT)$_{32}$ was heated with 200 μL 4-MOBT at 60 °C for 5 hours. The resulting mixture was washed with excess methanol and further purified by PTLC using a mixture of DCM and methanol (14:1) as the developing solvent.

3. Preparation of $Au_{52}(SR)_{32}$ /PMMA solid thin films

Uniform solid thin films for all four NC/PMMA samples were prepared by the spin-casting method. To make the PMMA stock solution, 40 mg of PMMA was dissolved in 1 ml of DCM (Solution A), and four different stock solutions of the four NCs were prepared by dissolving 1 mg of each NC in 1 ml of DCM (Solutions B_1, B_2, B_3, B_4). Next, 10 μL as-prepared solution 'A' was mixed with 20 μL of the as-prepared

solutions ‘B_1, B_2, B_3, B_4’ separately to obtain four ink-like solutions of the NC/PMMA mixture. Finally, the resulting mixture solutions were spin-coated one by one onto quartz plates, by taking 30 μL of the mixture solution and using 100 rpm rotation speed for 1 min 30 sec, which were subsequently air dried at room temperature.

4. Steady-state UV-Vis-NIR measurement

UV–vis–NIR spectra of all the Au NCs were collected on a UV-3600 Plus spectrophotometer (Shimadzu, range: 185-3300 nm). The cryogenic absorption measurements used a home-built low-temperature system, including the UV-3600 Plus spectrophotometer, a vacuum pump, an Optistat CF2 cryostat (Oxford Instruments), and a temperature controller.

5. Steady-State and Time-Resolved Photoluminescence and Cryogenic Measurements

Steady-state photoluminescence spectra were measured on an FLS-1000 spectrofluorometer (Edinburgh). PL lifetimes were measured by time-correlated single photon counting (TCSPC) on the same instrument. Visible PL was measured using a photomultiplier (PMT) as the detector. Near-infrared PL was measured using a wide-range InGaAs detector (600-1600 nm) cooled to -80 °C with liquid nitrogen. The cryogenic PL measurements used a home-built low-temperature system, including the FLS-1000 spectrofluorometer, a vacuum pump, an Optistat CF2 cryostat (Oxford Instruments), and a temperature controller. The PLQY of Au NCs in toluene and DCM were determined by an integrating sphere. The PLQY of Au NCs/PS film was determined by comparising the emission intensity with that of the solution.

6. Total Internal Reflection Fluorescence (TIRF) Microscopy Measurements

Investigation on the photostability of the NC emitters in the air at a very dilute concentration was done by Total Internal Reflection Fluorescence (TIRF) Microscopy using Olympus PlanApo 60X oil immersion objective with a numerical aperture 1.4 on an inverted microscope (Olympus IX-71). The laser excitation wavelength used was 488 nm output of an Ar-Kr laser (Coherent) using a CCD camera (Photometrics Evolve 512 EMCCD) to collect signal from the sample and a 780 nm long pass filter was used to minimize the scatterings from the samples. To track the intensities of the four different NC emitters (Au_{52}(*p*-MBT)$_{32}$, Au_{52}(4-EBT)$_{32}$, Au_{52}(I-PBT)$_{32}$, Au_{52}(TBBT)$_{32}$), first equally concentrated nM level stock solutions of all the four samples were freshly prepared using tetrachloroethene as a solvent. The samples for the microscopy were then prepared by spin-coating 60 μL of the stock solutions on glass coverslips using 4000 rpm rotation speed for 30 seconds, followed by air drying for 10 min at room temperature and imaged at the excitation wavelength of 488 nm using a continuous laser pulse with an acquisition time of 80 ms, a 2.0 ND (Neutral-Density) filter and an excitation power of 30 μW. A continuous sequence of images for all the NC samples was captured, consisting of 120 frames with no time lag, resulting in a 12-second movie. The images were analyzed using Image-Pro Plus software while maintaining the same display range for all four samples. A region of interest (ROI) with 67 pixels-square (0.18 μm/pixel, ~ 12 × 12 μm^2) was selected for intensity tracking measurement. Ten equally small 4 pixels-square regions (0.72 × 0.72 μm^2) consisting of the emitters were selected from the ROI and their maximum initial intensities were measured using the Image Pro Plus software for all four samples. The same technique was followed to get the background intensities surrounding the emitters from the ROI which were then subtracted from each frame to get the actual intensity of the emitters for all the samples. The final data were then plotted in terms of the percentage distribution of objects (emitters) vs their intensities to get the most probable initial intensity from the distribution of ten emitters for each sample. Then one of the emitters with the most probable initial intensity was selected from each sample and its intensity over time was tracked and plotted to compare the single-step photobleaching time for the four $Au_{52}(SR)_{32}$ NCs.

7. X-ray crystallography

A black columnar crystal of $Au_{52}(S\text{-}p MeC_6H_4)_{32}$ was used for data collection on a Bruker Duo diffractometer with a PHOTON II detector and Iμs CuKα radiation (1.54178 Å) at 200 K. The structure was solved in the monoclinic space group P2(1)/c. Integration and scaling of the data yielded 53,5238 reflections, of which 31,983 were unique (and 16,111 unique data with $I > 2\sigma(I)$), to a maximum of θ = 50.6° (d = 1.0 Å) with a completeness of 99.5% and an R_{int} of 16.22%.
All Au and S atoms were refined with anisotropic thermal displacement parameters, while all carbon atoms were treated with an isotropic thermal displacement parameter and subjected to similarity restraint (SIMU). Hydrogen atoms were placed in idealized positions and treated with a riding model. Each carbon atom belonging to a phenyl ring was constrained at the corners of a regular hexagon (AFIX 66), numerous distance restraints (SADI) were applied as needed, coplanarity restraints were also applied as needed (FLAT). Thus, the final model consisted of 1,301 parameters with 269 restraints.

The final refinement on F^2 converged at R_1 = 12.85% ($I > 2\sigma(I)$) and wR_2 = 39.17% (all data). The goodness-of-fit was 1.814. The largest residual density extrema were 3.919 and –2.140 ($e^-/Å^3$) with an RMS deviation of 0.525 $e^-/Å^3$.

8. Absolute Quantum Yield Determination

The absolute PLQY of $Au_{52}(p\text{-MBT})_{32}$ in DCM solution at room temperature was determined by an integsrating sphere. A sample figure is shown below. For such a measurements, the regular sample holder is removed, and the integrating sphere is mounted in the FLS1000 spectrometer. The blank spectrum (**light blue line** in the Figure) was obtained by putting a blank in the integrating sphere and collecting the emission scan (repeat 5 times) from 500 nm to 1600 nm with 470 nm excitation. The sample spectrum (**yellow**) was measured by replacing the blank with a dilute **$Au_{52}(p\text{-MBT})_{32}$** solution (0.1 OD at 470 nm) and repeating the emission scan (5 times) from 500 nm to 1600 nm with 610 nm excitation. The PLQY is calculated by:

$$\eta = L_{sample} \,/\, E_{blank} - E_{sample}$$

where, E_{blank} is the area under the blue line between 500 and 530 nm, E_{sample} is the area under the yellow line between 500 and 530 nm, L_{sample} is the area under the yellow line between 800 and 1200 nm.

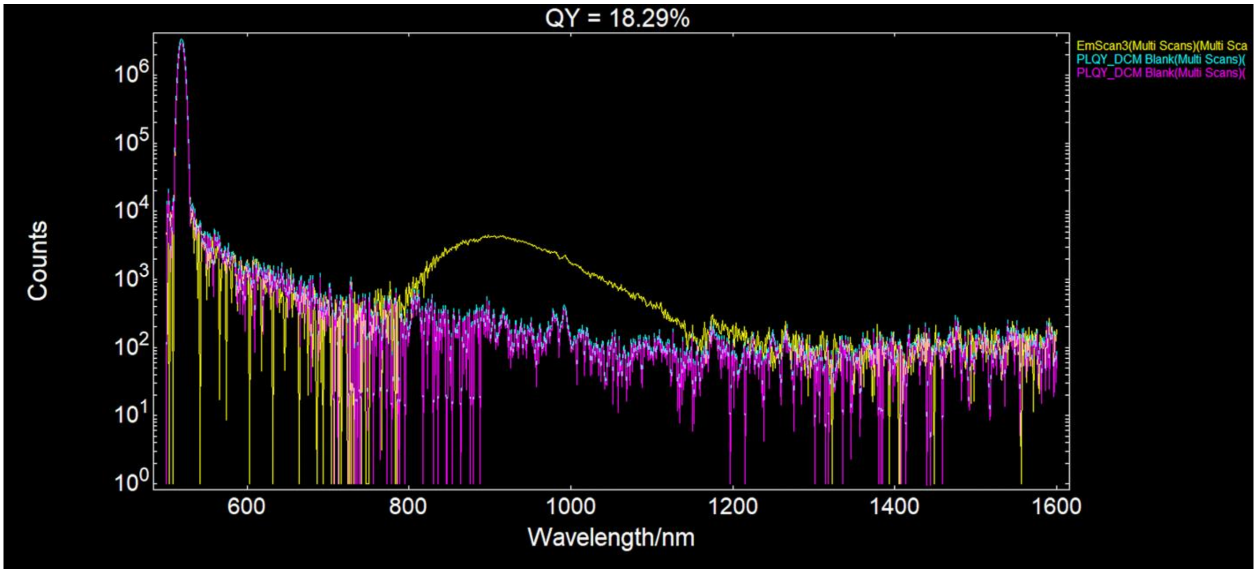

9. Relative Quantum Yield Determination

The relative quantum yield (Φ_S) of the sample is calculated by using:

$$\Phi_{\mathrm{S}} = \Phi_{\mathrm{R}}(\frac{I_S}{I_R})(\frac{1-10^{-A_R}}{1-10^{-A_S}})(\frac{n_S}{n_R})^2$$

where, Φ_{R} is the quantum yield of the reference (standard), I is the integrated PL intensity, A is the absorbance of the solution at the excitation wavelength, n is the refractive index of the solvent, and the subscripts (S and R) represent the sample and the reference, respectively.

Supporting Figures:

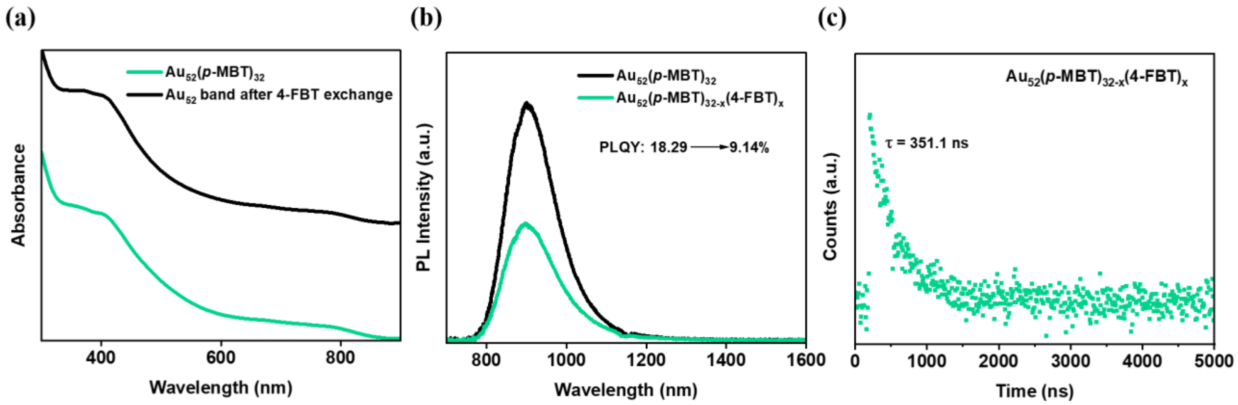

Figure S1. (a) UV-vis absorption spectra of **Au_{52}(*p*-MBT)$_{32\text{-}x}$(4-FBT)$_x$**. (b) PL spectra of **Au_{52}(*p*-MBT)$_{32\text{-}x}$(4-FBT)$_x$** in tetrachloroethene under helium atmosphere and O_2 atmosphere (excitation wavelength: 470 nm). (c) The corresponding PL decay profile.

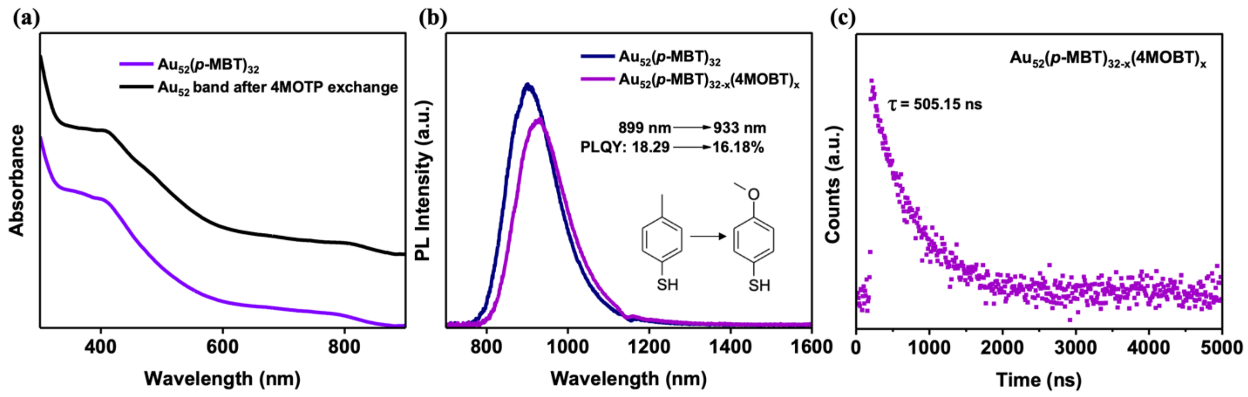

Figure S2. (a) UV-vis absorption spectra of **Au_{52}(*p*-MBT)$_{32\text{-}x}$(4-MOBT)$_x$**. (b) PL spectra of **Au_{52}(*p*-MBT)$_{32\text{-}x}$(4-MOBT)$_x$** in tetrachloroethene under helium atmosphere and O_2 atmosphere (excitation wavelength: 470 nm). (c) The corresponding PL decay profile.

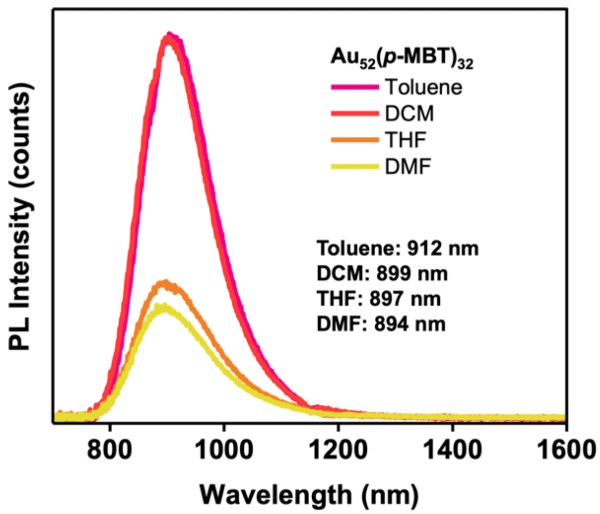

Figure S3. PL spectra of **Au_{52}(*p*-MBT)$_{32}$** in different solvents (PL intensities: Tol.~DCM > THF >DMF).

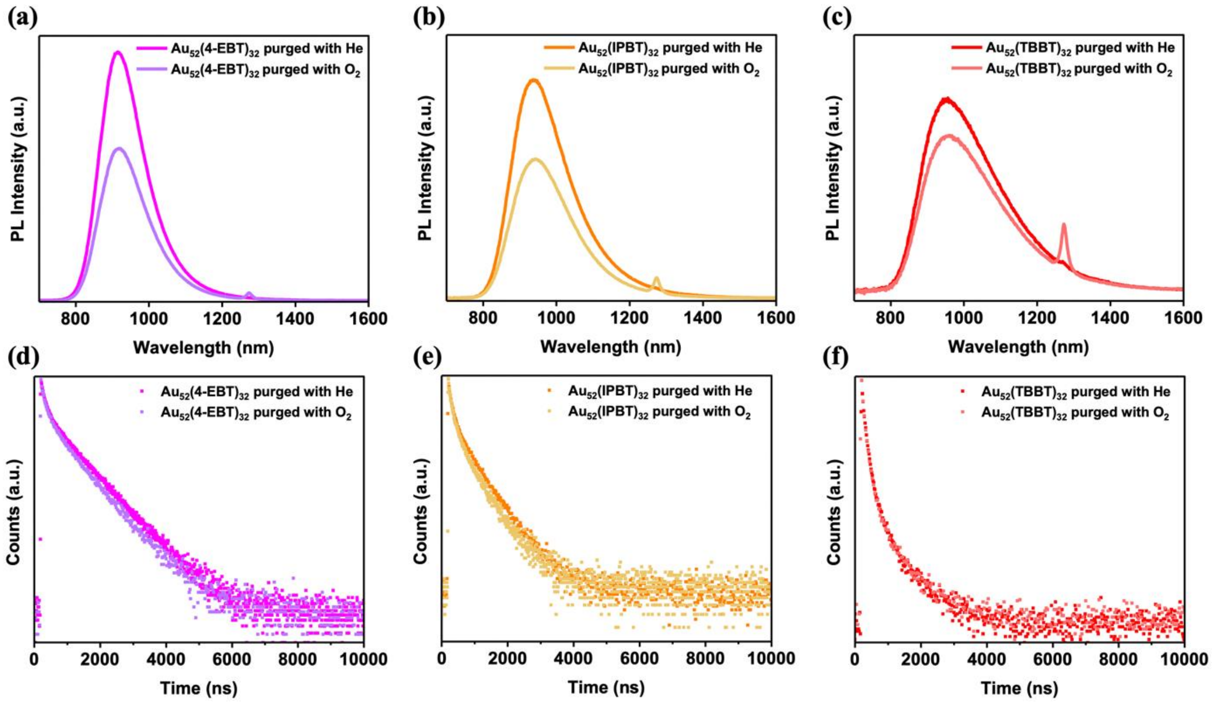

Figure S4. (a)-(c) PL spectra of **Au_{52}(4-EBT)$_{32}$**, **Au_{52}(IPBT)$_{32}$** and **Au_{52}(TBBT)$_{32}$** in tetrachloroethene (helium and O_2 atmosphere, respectively; the $^1O_2$ phosphorescence peak is observed at 1274 nm (sharp peak) riding on the Au_{52} PL curves). (d)-(e) the corresponding PL decay profiles.

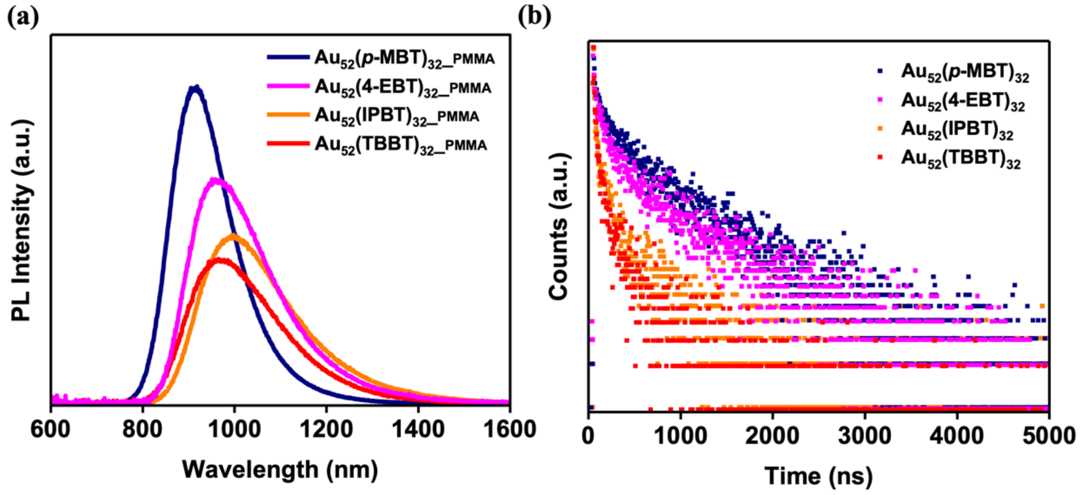

Figure S5. (a) PL spectra of the four $Au_{52}(SR)_{32}$ films and **(b)** their PL lifetime profiles. Both trends are similar to those in solution, see data summary in Table 2 (main text).

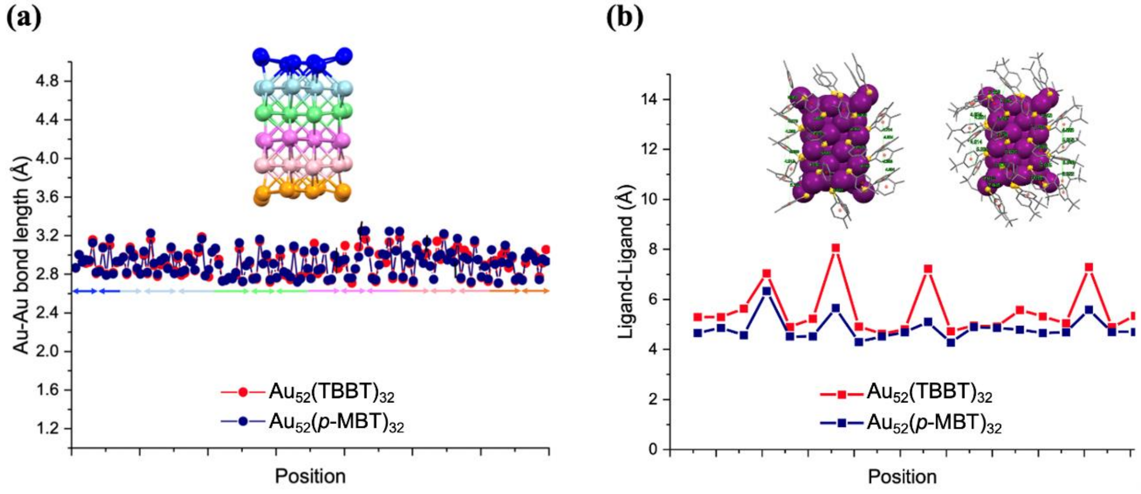

Figure S6. Comparison of distances of **$Au_{52}(p\text{-}MBT)_{32}$** and **$Au_{52}(TBBT)_{32}$** X-ray structures, a) Au-Au bond lengths (no discernable difference), and b) inter-ligand distances (for simplicity, only the face-to-face packed ligands on (100) facets are selected for comparison; such distances in $Au_{52}(p\text{-}MBT)_{32}$ are much smaller compared to the –TBBT case).

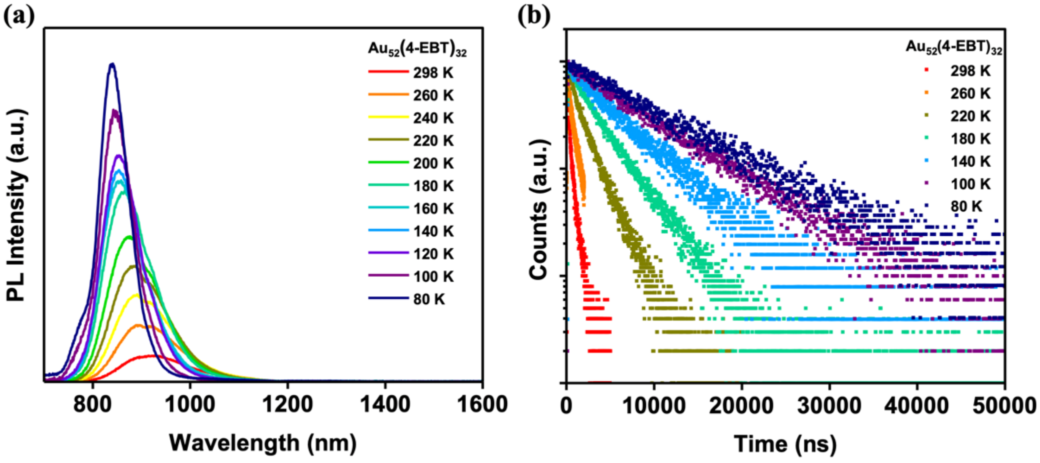

Figure S7. (**a**) PL spectra of **Au_{52}(4-EBT)$_{32}$** at different temperatures and (**b**) PL lifetime profiles.

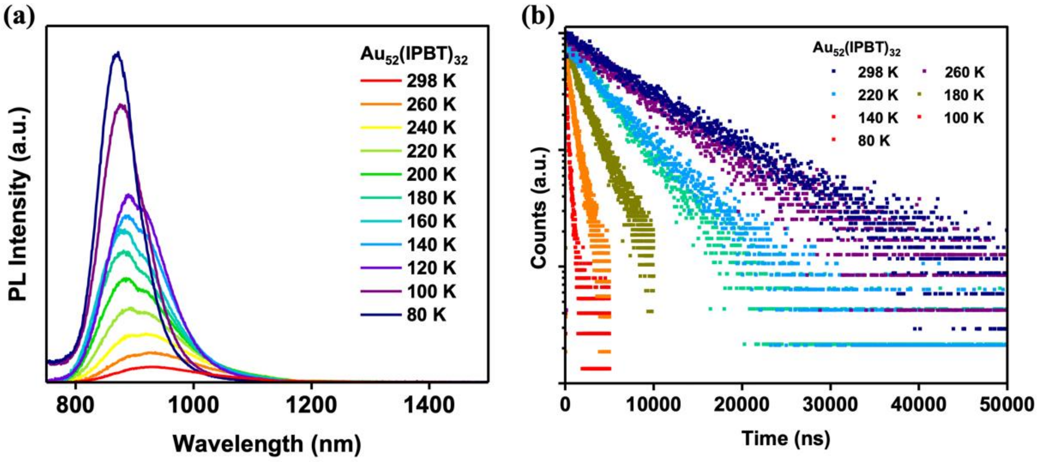

Figure S8. (**a**) PL spectra of **Au_{52}(IPBT)$_{32}$** at different temperatures and (**b**) PL lifetime profiles.

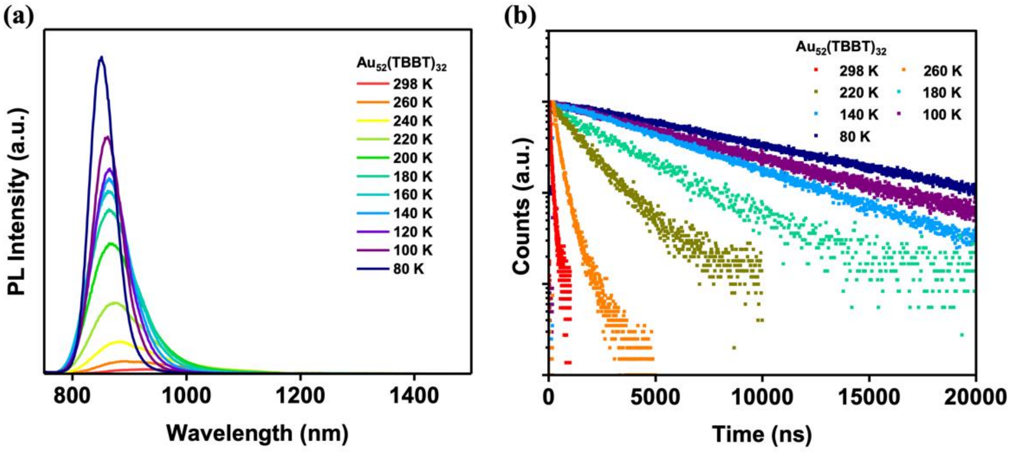

Figure S9. (**a**) PL spectra of **Au_{52}(TBBT)$_{32}$** at different temperatures and (**b**) PL lifetime profiles (see S7-S9 data fitting in Fig S10, the case of *p*-MBT in presented in main text).

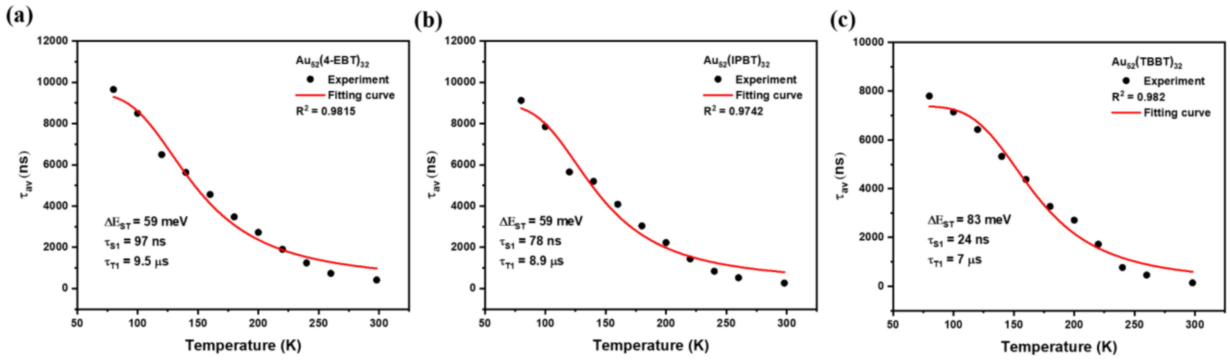

Figure S10. Plots of emission lifetime collected at 470 nm excitation against temperature for the cases of 4-EBT, IPBT and TBBT (red curves: data fitting by **Eq 2** (see the equation in main text), the case of *p*-MBT is presented in main text).

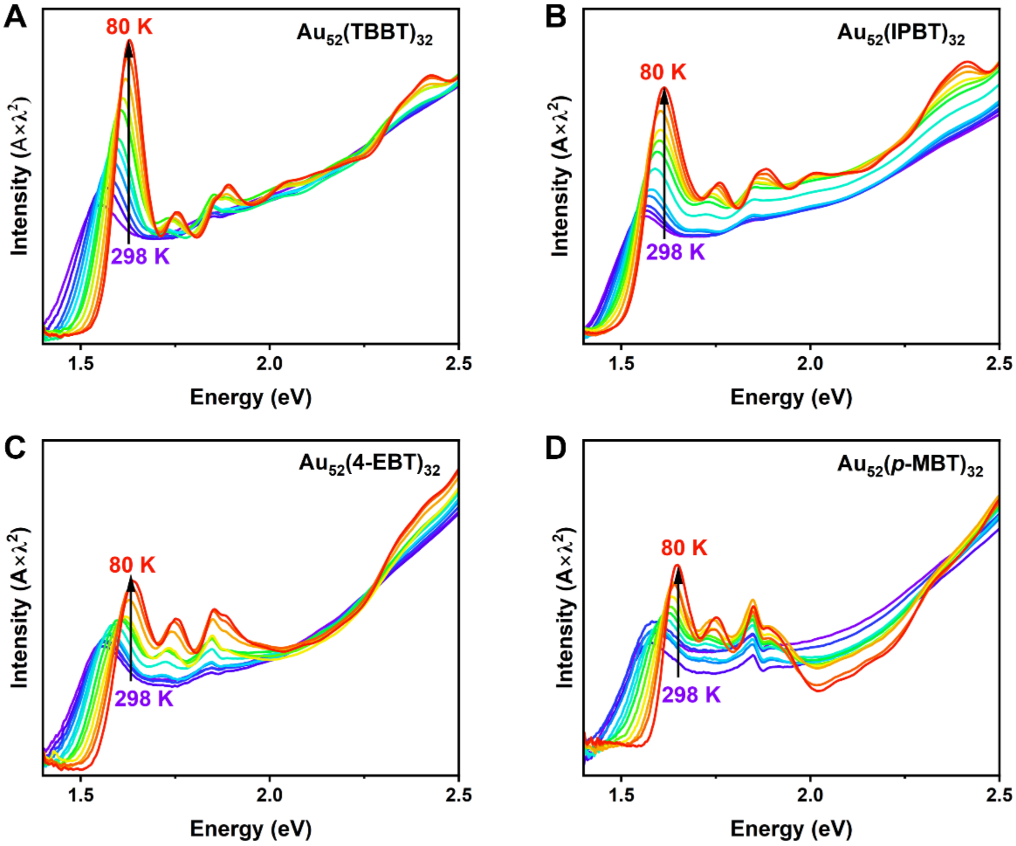

Figure S11. Cryogenic optical absorption spectra at different temperatures of (A) **$Au_{52}(TBBT)_{32}$**, (B) **$Au_{52}(IPBT)_{32}$**, (C) **$Au_{52}(4\text{-}EBT)_{32}$**, (D) **$Au_{52}(p\text{-}MBT)_{32}$** dissolved in 2-methyltetrahydrofuran (for clear 'glass' formation at low temperatures).

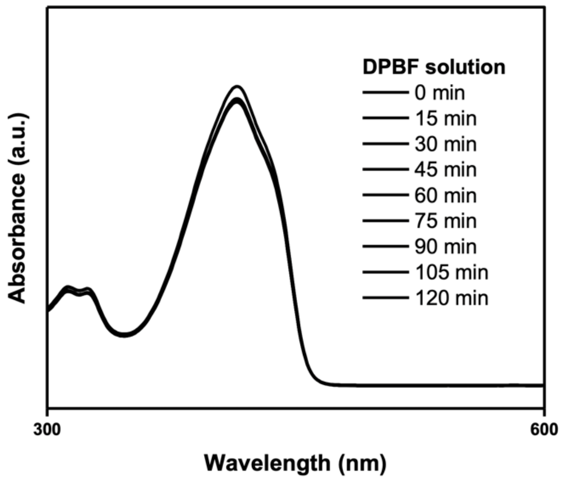

Figure S12. Blank DPBF solution in DMF in the absence of gold nanoclusters under irradiation with λ = 500 nm.

Table S1. Sample and crystal data for Au_{52}(*p*-MBT)$_{32}$.

**Chemical formula**	$C_{224}H_{224}Au_{52}S_{32}$	
**Formula weight**	14184.20 g/mol	
**Temperature**	200(2) K	
**Wavelength**	1.54178 Å	
**Crystal size**	0.067 x 0.075 x 0.228 mm	
**Crystal system**	monoclinic	
**Space group**	$P2_1/c$	
**Unit cell dimensions**	a = 29.0624(16) Å	α = 90°
	b = 38.055(2) Å	β = 96.066(3)°
	c = 27.7450(18) Å	γ = 90°
**Volume**	30513.(3) Å^3	
**Z**	4	
**Density (calculated)**	3.088 g/cm^3	
**Absorption coefficient**	47.954 mm^{-1}	
**F(000)**	24752	
**Theta range for data collection**	1.92 to 50.57°	
**Index ranges**	-28<=h<=29, -35<=k<=37, -27<=l<=27	
**Reflections collected**	535238	
**Independent reflections**	31983 [R(int) = 0.1622]	
**Coverage of independent reflections**	99.5%	
**Absorption correction**	Numerical Mu From Formula	
**Max. and min. transmission**	0.1410 and 0.0320	
**Refinement method**	Full-matrix least-squares on F^2	
**Refinement program**	SHELXL-2019/1 (Sheldrick, 2019)	
**Function minimized**	Σ w(F_o^2 - F_c^2)^2	
**Data / restraints / parameters**	31983 / 269 / 1301	
**Goodness-of-fit on F^2**	1.814	
**Final R indices[$I>2\sigma(I)$]**	R1 = 0.1285, wR2 = 0.3457	
**R indices (all data)**	R1 = 0.2243, wR2 = 0.3917	
**Largest diff. peak and hole**	3.919 and -2.140 eÅ^{-3}	
**R.M.S. deviation from mean**	0.525 eÅ^{-3}	

Reference:

(s1) Zhuang, S.; Liao, L.; Li, M.-B.; Yao, C.; Zhao, Y.; Dong, H.; Li, J.; Deng, H.; Li, L.; Wu, Z. The fcc Structure Isomerization in Gold Nanoclusters. *Nanoscale* **2017**, *9*, 14809-14813.
